# Triggered integer charge transfer: energy-level alignment at an organic-2D material interface†

**DOI:** 10.1039/d4na00462k

**Published:** 2024-07-30

**Authors:** Maximilian Schaal, Anu Baby, Marco Gruenewald, Felix Otto, Roman Forker, Guido Fratesi, Torsten Fritz

**Affiliations:** a Institute of Solid State Physics, Friedrich Schiller University Jena Helmholtzweg 5 07743 Jena Germany torsten.fritz@uni-jena.de; b Department of Materials Science, University of Milano-Bicocca Via R. Cozzi 55 20125 Milano Italy; c STMicroelectronics Via Tolomeo 1 20010 Cornaredo Italy; d ETSF and Dipartimento di Fisica “Aldo Pontremoli”, Università degli Studi di Milano Via Celoria, 16 20133 Milano Italy

## Abstract

Weakly interacting systems such as organic molecules on monolayers of hexagonal boron nitride (h-BN) offer the possibility of single integer charge transfer leading to the formation of organic ions. Such open-shell systems exhibit unique optical and electronic properties which differ from their neutral counterparts. In this study, we used a joint experimental and theoretical approach to investigate the charge transfer of perylene-3,4,9,10-tetracarboxylic dianhydride (PTCDA) molecules on h-BN/Ni(111) by using differential reflectance spectroscopy (DRS), scanning tunneling spectroscopy (STS), and photoelectron orbital tomography (POT) measurements in combination with density functional theory (DFT) calculations. Our results show that the PTCDA monolayer consists of highly ordered organic radical anions and neutral molecules. In addition, the occurrence of the integer charge transfer is discussed based on the energy-level alignment. Since the integer charge transfer is not limited to PTCDA, we propose that the h-BN covered Ni(111) surface is a promising substrate for studying the optical and electronic properties of highly ordered organic anions.

## Abstract 2

Weakly interacting systems such as organic molecules on monolayers of hexagonal boron nitride (h-BN) offer the possibility of single integer charge transfer leading to the formation of organic ions.

## Graphical abstract

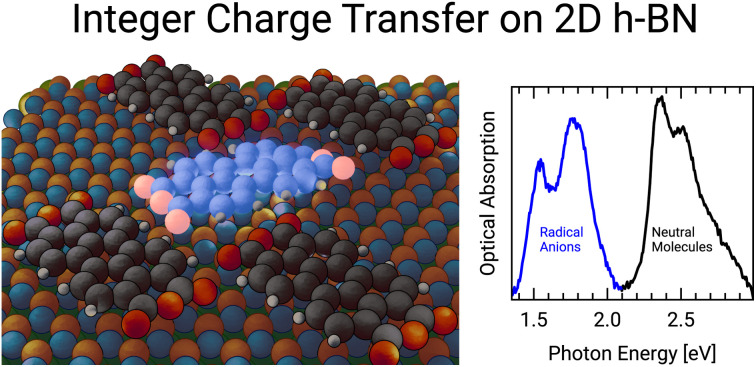

## Introduction

Two-dimensional (2D) materials like graphene and hexagonal boron nitride (h-BN) are potential candidates for the decoupling of organic molecules from metal substrates.^1,2^ These atomically thin layers are of great interest because of their possibility to regain the intrinsic molecular properties such as the typically narrow optical absorption and/or emission in contrast to the adsorption on the bare metal substrate.^3^ Especially, 2D h-BN is quite interesting because of its large band gap of approx. 6 eV.^4^ It can be prepared with a low defect density.^5^ Additionally, weakly interacting substrates can show integer charge transfer by the adsorption of organic molecules^6–12^ if the work function of the substrate is lower than the electron affinity or larger than the ionization energy of the organic molecules. The simplest integer charge transfer process is the formation of molecular anions or cations by adding or removing one electron from the organic molecules, respectively. These systems which have one unpaired electron in the highest occupied molecular orbital (HOMO) are also called open-shell systems and have unique optical, electronic and chemical properties which differ from their neutral counterparts. For example, anions and cations have a reduced optical and electronic gap as well as a low ionization energy or high electron affinity, respectively.^13^ The latter is especially interesting for the use as electron donors or acceptors in novel organic devices^14^ and can open new pathways to chemical reactions on 2D materials.^15^ In addition, such open shell molecules are important for spintronic applications^16,17^ (organic quantum bits) and molecular magnets.^18^

**Fig. 1:**
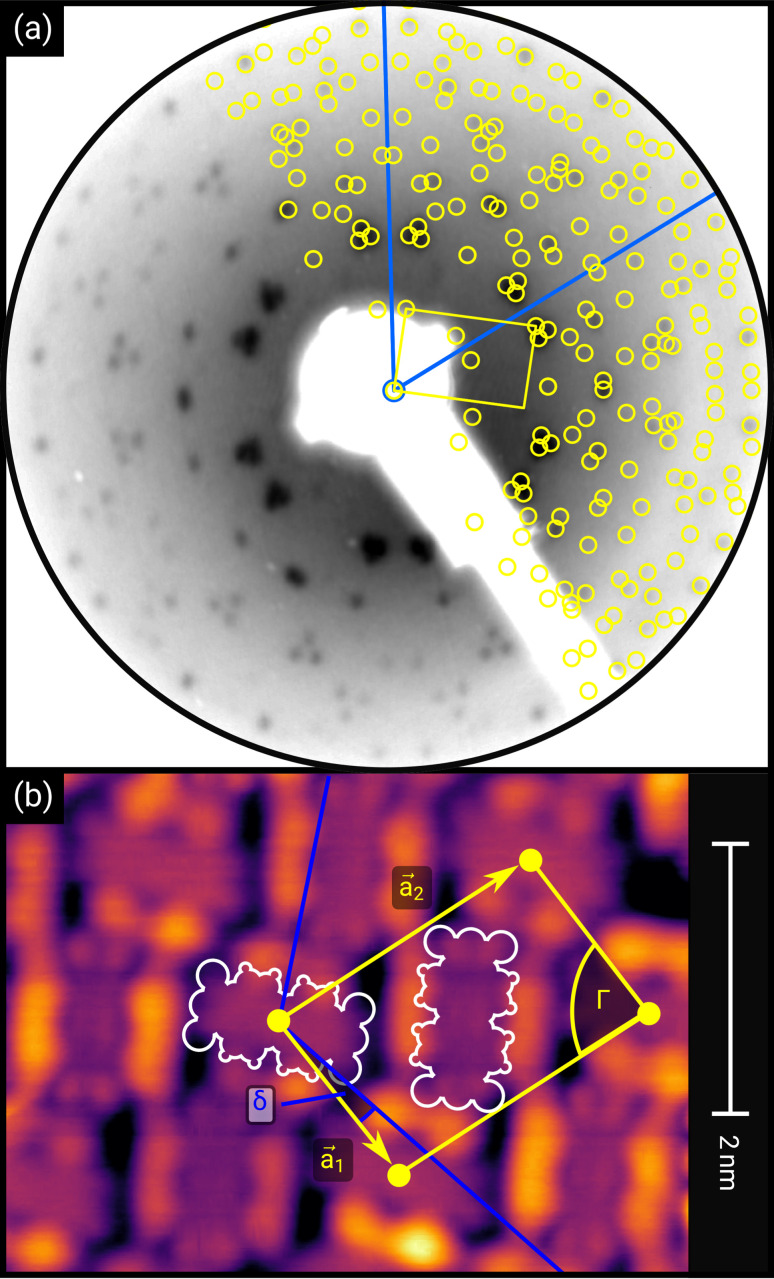
(a) Distortion-corrected LEED image (*E* = 22.0 eV, logarithmic intensity scale, contrast-inverted) of 1.5 MLE PTCDA on h-BN/Ni(111). Half of the LEED image is superimposed by the LEED simulation (best numerical fit). Yellow points and lines correspond to the reciprocal lattice of the PTCDA structure including rotational and mirror domains. Blue lines indicate two primitive reciprocal lattice directions of the substrate. The lattice parameters are summarized in Table 1. The inset shows the chemical structure of PTCDA. (b) STM image (*V*_B_ = +0.1 V, *I*_t_ = 10 pA, *T* = 4.5 K) of the same sample, superimposed by the real-space structure of the molecular lattice (marked in yellow) as well as white contours of the two molecules in the unit cell. Blue lines indicate the direction of the primitive lattice vectors of the substrate.

Different kinds of charge transfer processes have been reported for organic molecules on h-BN monolayers such as temperature-dependent charge transfer,^19^ site-selective charge transfer^20^ and tip-induced charge transfer.^21^ In this study we investigate the charge transfer process of highly ordered perylene-3,4,9,10-tetracarboxylic dianhydride (PTCDA, C_24_H_8_O_6_, CAS No.: 128-69-8, chemical structure see inset in Fig. 1(a)) layers on h-BN/Ni(111). Due to the very small lattice mismatch of 0.4%^4^ and therefore flat morphology,^5^ a monolayer of h-BN on Ni(111) was chosen as substrate which favors the growth of 2D highly ordered organic layers.^3^ By means of differential reflectance spectroscopy (DRS), photoelectron spectroscopy (PES), and scanning tunneling spectroscopy (STS) measurements we observe that this system consists of both negatively charged and neutral molecules. Furthermore, the lateral structure is investigated by quantitative low-energy electron diffraction (LEED) and scanning tunneling microscopy (STM). The observed charge transfer is supported by density functional theory (DFT) calculations. Finally, we develop a theoretical model based on the energy-level alignment to explain the origin of the charge transfer. We find that the integer charge transfer occurs because the electron affinity of the molecular layer is larger than the work function of the substrate in combination with a weak molecule–substrate interaction of PTCDA on h-BN/Ni(111).

## Experimental methods and calculation details

The PTCDA molecules were purchased from Sigma-Aldrich and purified by temperature gradient sublimation^22^ as well as thoroughly degassed in ultra-high vacuum (UHV). The Ni(111) single crystal was purchased from MaTecK GmbH with a nominal purity of 99.99%. The preparation of the nickel single crystal, the PTCDA deposition as well as all experimental techniques are performed in UHV with a base pressure smaller than 5 × 10^−10^ mbar. A clean surface was prepared by several cycles of Ar^+^ sputtering and annealing at 850 °C until the C 1s and O 1s signal in XPS was below the detection threshold. The growth of an h-BN monolayer on Ni(111) was achieved by using chemical vapor deposition (CVD). In doing so, borazine was used as precursor and was deposited onto the Ni(111) surface at a substrate temperature of 850 °C similar to ref. 5 and 23. This results in a continuous, flat^5^ and self-limited^23^ h-BN monolayer. The borazine was purchased from Katchem spol. s. r. o. with a nominal purity larger than 97%. *In situ* DRS was measured during the PTCDA deposition onto h-BN/Ni(111) utilizing a 100 W halogen tungsten lamp, a blazed-grating monochromator (Acton Research SpectraPro SP2156), and a thermoelectrically cooled charge-coupled device (CCD) (Princeton Instruments PIXIS 100BR eXcelon/UV).^24–26^ The DRS signal is defined as:
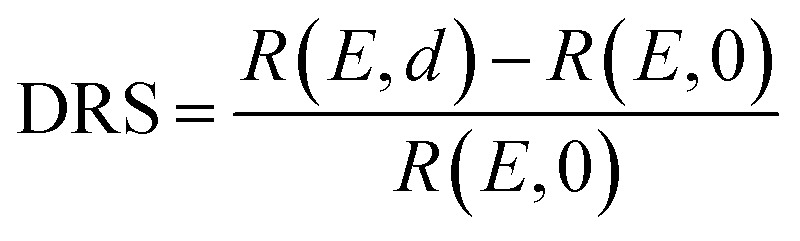
where *R*(*E*, 0) is the reflectance of the substrate and *R*(*E*, *d*) the reflectance of the substrate covered by an adsorbate with a thickness *d*. Furthermore, changes between two consecutive spectra are better visible by calculating the ΔDRS signal.
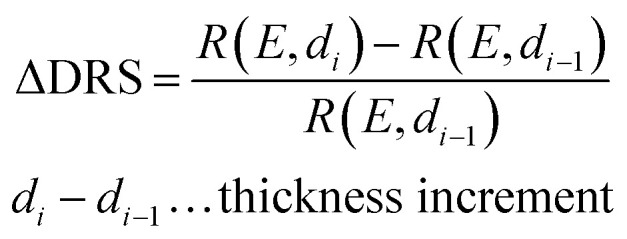


The lateral structure was investigated on the one hand by low energy electron diffraction (LEED) recorded by using an Omicron MCP-LEED (MCP2-SPECTALEED) and on the other hand by scanning tunneling microscopy (STM) recorded by using a JT-STM/AFM (SPECS Surface Nano Analysis GmbH) with a tungsten tip operated at 4.5 K. The LEED images were distortion corrected by using the freely available software LEEDCal.^27^ Quantitative LEED analysis was performed with the software LEEDLab (software is available upon reasonable request from the corresponding author). The local electronic structure was investigated by scanning tunneling spectroscopy (STS) using the already mentioned STM apparatus. The area-averaged electronic and chemical structures were investigated by means of X-ray and ultraviolet photoelectron spectroscopy (XPS and UPS). For those measurements monochromatized Al Kα (SPECS Focus 500, *E*_excitation_ = 1486.71 eV), monochromatized and p-polarized He Iα (SPECS UVLS-600, *E*_excitation_ = 21.22 eV) radiation, and a SPECS PHOIBOS 150 hemispherical electron analyzer equipped with a 3D delay line detector (SPECS DLD4040-150). Density functional theory (DFT) calculations of single molecules in vacuum as well as in a polarizable medium were performed with the Gaussian 16 code^28^ using the B3LYP exchange correlation functional and the 6-311G++(d,p) basis set. The ionization energy (IE) and the electron affinity (EA) were calculated by means of the ΔSCF approach.^29^IE = *E*_tot_(neutral PTCDA) − *E*_tot_(cationic PTCDA)EA = *E*_tot_(anionic PTCDA) − *E*_tot_(neutral PTCDA)

Molecular orbitals of a single PTCDA molecule in vacuum were used for the POT simulations. DFT calculations for molecules adsorbed on h-BN/Ni(111) were performed by the Quantum-ESPRESSO package^30,31^ that adopts periodically repeated cells, ultrasoft pseudopotentials and plane waves. We have chosen the vdW-DF2-C09 exchange–correlation functional^32,33^ that includes dispersion forces. For pseudopotentials, we used those from pslibrary^34^ with plane wave cutoffs set to 47 Ry and 326 Ry for the wavefunctions and electron density, respectively. The model of the Ni(111) surface consists of a slab with three Ni layers, with h-BN positioned at the adsorption sites (N, B) = (top, fcc).^35^ Molecules were placed on top, and their coordinates were optimized together with those of h-BN and surface Ni atoms until forces were lower than 1 mRy per bohr. A lateral superstructure with cell vectors 12.4 Å and 19.6 Å is constructed, following our LEED results. Since this unit cell does not match perfectly the Ni periodicity which is a requirement for the simulations, we constructed a 
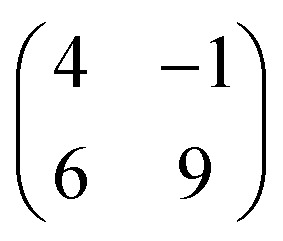
 supercell, having 42 surface Ni atoms (286 atoms overall) whose cell vectors are stretched (5% and 8%) to the experimental dimensions. In this way we preserve the correct periodicity of the organic overlayer as we did in previous work.^36^ A complete relaxation of the two PTCDA molecules in the unit cell was realized, including h-BN and the topmost Ni(111) layer. We sample the Brillouin zone by a Monkhorst–Pack^37^ 3 × 2 shifted mesh. In the perpendicular direction, a vacuum space of 20 Å separates molecules from the replicated Ni slab.

## Results and discussion

### Thin film growth and structural analysis

First, we start with the discussion of the lateral structure of a PTCDA monolayer on h-BN/Ni(111). This will be the foundation of our DFT calculations as well as of the simulation of the photoelectron momentum maps (PMMs). During the growth of the thin film we used DRS to determine the layer thickness by investigating changes in the optical properties of the organic thin film (see Fig. S1 in the ESI†). By increasing the coverage, we observe at a certain point that the signal of the features at 1.5 eV and 1.8 eV do not increase anymore. We use this specific point to define one monolayer equivalent (MLE) of PTCDA on h-BN/Ni(111). The LEED pattern of approx. 1 MLE PTCDA on h-BN/Ni(111) is shown in Fig. S2.† Instead of distinct diffraction spots, azimuthally stretched intensity distributions are observed which are caused by rotational disorder which is often observed for submonolayer coverage.^38,39^ This arises from the fact that our definition of 1 MLE is based on changes of the optical properties of the molecules due to the adsorption of molecules in the second layer. However, the starting adsorption of molecules in the second layer does not automatically mean that the first layer is completely filled. This fact becomes even more obvious by looking at the LEED pattern of a sample with a slightly higher coverage of approx. 1.5 MLE which is depicted in Fig. 1. For this sample we observe distinct diffraction spots and no hints for ring-like patterns. The investigation of the lateral structure of 1.5 MLE PTCDA on h-BN/Ni(111) was realized by a quantitative distortion-corrected LEED analysis and LT-STM measurements. Fig. 1 shows the LEED image superimposed by the best-fit geometric LEED simulation.^27,40^ The corresponding lattice parameters and epitaxy matrix are summarized in Table 1. By comparing our structural model of PTCDA on h-BN/Ni(111) with the lateral structure of 1.0 MLE PTCDA on BlueP/Au(111)^41^ we find very good agreement. Even the angle between the substrate and the adsorbate lattice (domain angle) matches very well. Noteworthily, PTCDA grows epitaxially on noble metal single crystals like Au(111)^42^ and Ag(111)^36^ with almost the same adsorbate lattice and forms the well-known herringbone structure of flat-lying PTCDA molecules, but with different domain angles. An insight into the unit cell composition was achieved by the high-resolution STM image in Fig. 1, which shows the two azimuthally rotated molecules per unit cell and therefore confirms the herringbone structure. Next, we use the projection method proposed by Forker *et al.*^43^ to search for possible coincidences between the adsorbate and the substrate lattices. We find an on-line coincidence of the order (*h*_s_, *k*_s_) = (0, 1) : (*h*_a_, *k*_a_) = (−1, 9) within the error margins of the epitaxy matrix elements. Note that in addition to these individual uncertainties there is an absolute scaling uncertainty of ±1% for the entire matrix. The corresponding refined matrix is also provided in Table 1.

**Table 1:** Lattice parameters obtained from our LEED analysis and of the refined lattice parameters considering an on-line epitaxy of the order (*h*_s_, *k*_s_) = (0, 1) : (*h*_a_, *k*_a_) = (−1, 9) (Laue indices of the substrate and adsorbate lattice). *Γ* is defined as the angle between the lattice vectors of the adsorbate 
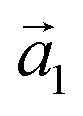
 and 
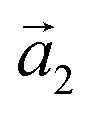
. The angle between the adsorbate lattice vector 
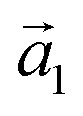
 and the direction of the substrate lattice vector 
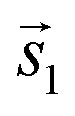
 is labeled with *δ*. The uncertainty of the numerical fitting procedure (single standard deviation) is given in parentheses behind each value and refers to the last significant digits. The refined matrix was achieved by changing the entries within the error margin by taking into account the scaling as well as the individual numerical error

	1.5 MLE PTCDA on h-BN/Ni(111)	Refined matrix (*h*_s_, *k*_s_) = (0, 1) (*h*_a_, *k*_a_) = (−1, 9)
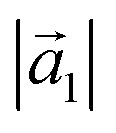 (Å)	12.40(3)	12.4
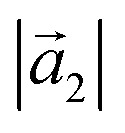 (Å)	19.64(4)	19.7
*Γ* (°)	90.8(2)	91.0
*δ* (°)	−10.0(1)	−10.0
Epitaxy matrix	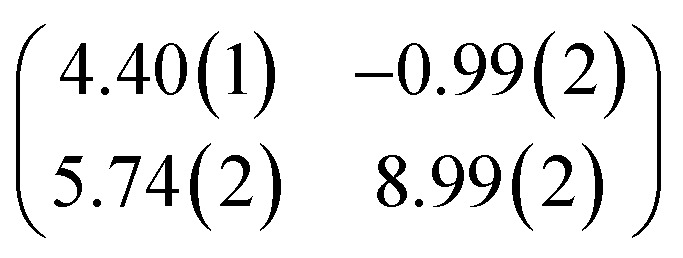	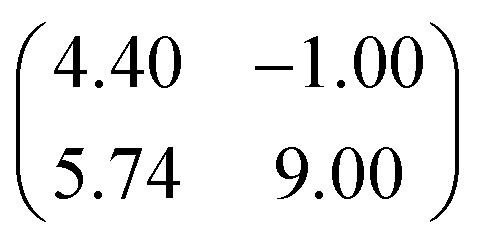

### Optical spectroscopy – investigating the coupling strength to the substrate

**Fig. 2:**
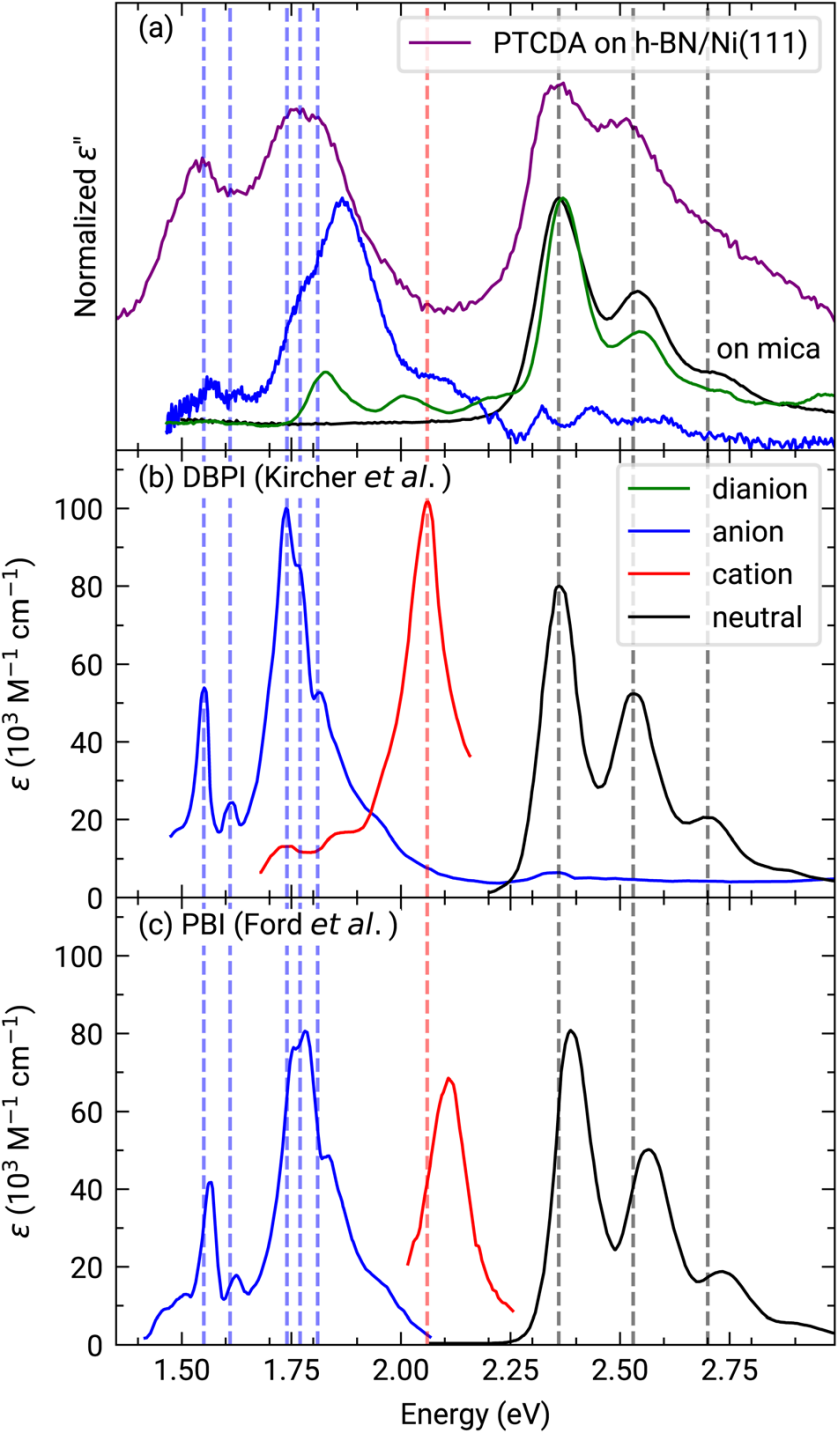
(a) Experimentally obtained imaginary part of the dielectric function of approx. 1.0 MLE PTCDA on h-BN/Ni(111) (purple). Additionally, the graph shows the neutral monomer (black), anion (blue) and dianion (green) spectra obtained for PTCDA on mica (vertically shifted to enhance visibility).^44,45^ Each spectrum is normalized to the maximum of the associated spectroscopic features. (b) and (c) absorbance spectra of DBPI and PBI anions, cations (red) and neutral monomers in solution measured by Kircher *et al.*^46^ and Ford *et al.*,^47^ respectively. Adapted with permission (Copyright 1999, American Chemical Society and 1989, Royal Society of Chemistry). Vertical lines were added as guides to the eye.

The investigation of the optical properties of PTCDA on h-BN/Ni(111) was performed by measuring *in situ* DRS during the growth process. This method makes it possible to distinguish hybridized, decoupled as well as charged molecules.^3,8,24,44^ By using a numerical algorithm, we extracted the real and imaginary part of the dielectric function from the measured DRS signal.^24^ In the following, we will focus on the imaginary part of the dielectric function *ε*′′ only, which is depicted in Fig. 2(a), as this physical quantity resembles the optical absorption behavior. We also added the monomer spectrum as well as the anion and the dianion spectrum of PTCDA on mica as reference in Fig. 2(a).^45^ By comparing the optical absorption of PTCDA on h-BN/Ni(111) with the neutral monomer, anion and dianion spectrum, it is obvious that the monomer spectrum alone cannot explain the optical absorption of PTCDA on h-BN/Ni(111). Instead, the superposition of either the anion and dianion spectrum or monomer and anion spectrum matches quite well (when neglecting the different *ε*′′ magnitudes). Therefore, the measurements suggest that a charge transfer from the h-BN/Ni(111) substrate to the molecular thin film happens, which leads to a negative charging of some of the PTCDA molecules. Furthermore, we can also compare our measurements with absorption spectra of two diimide-derivatives of PTCDA namely, *N*,*N*′-bis(1-hexylheptyl)-3,4:9,10-perylenebis(dicarboximide) (PBI) and *N*,*N*′-bis(2,5-di-*tert*-butylphenyl)-3,4:9,10-perylenebis(dicarboximide) (DBPI). Both molecules differ only by the optically inactive side groups attached to each nitrogen atom, while their chromophores are practically identical to that of PTCDA. In fact, Kircher *et al.* already stated that the absorption and fluorescence spectra of these derivatives are very similar.^46^ This becomes also obvious by comparing the absorption spectra of the charged as well as neutral DBPI and PBI molecules (see dashed lines in Fig. 2(b) and (c)). Furthermore, the comparison to PTCDA on h-BN/Ni(111) also shows a very good agreement with the neutral and anionic species of DBPI and PBI. Therefore, we conclude that the PTCDA monolayer on h-BN/Ni(111) consists of anionic and neutral molecules. It is noticeable that the spectroscopic features of the PTCDA monolayer on h-BN/Ni(111) are broadened due to the non-negligible intermolecular and molecule–substrate interaction.

In order to emphasize the special nature of the here investigated interface we can compare with PTCDA on other 2D materials. In a previous work we observed that the optical constants of PTCDA on h-BN/Pt(111) closely resemble the monomer spectrum and that therefore PTCDA is decoupled from the metal surface by the h-BN interlayer.^2^ Recently, we published similar findings for a monolayer of PTCDA on a BlueP-Au-network on Au(111), where PTCDA is electronically decoupled from the metal support as indicated by the monomer behavior in our optical data.^41^ Thus, the here observed charge transfer between PTCDA and hBN/Ni(111) is particularly interesting and raises the following questions: is it possible to independently confirm this charge transfer by complementary measurements, and what is the difference between these 2D materials? We will answer each of these questions in the following sections.

### Electronic structure – manifesting the charge transfer

**Fig. 3:**
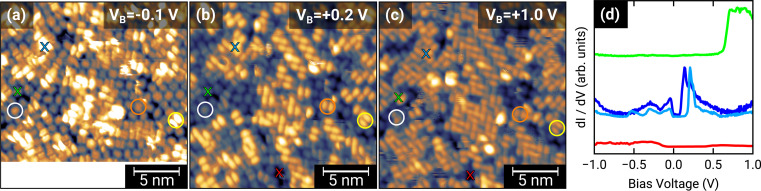
(a–c) 20 × 20 nm^2^ STM images of 1.0 MLE PTCDA on h-BN/Ni(111). The same scan region scanned with different bias voltages is displayed. All STM images were recorded with a tunneling current of 30 pA and at a temperature of 4.5 K. Colored crosses in the STM images indicate the location of the performed STS measurements which are displayed in (d), vertically shifted for the sake of clarity. Colored circles are a guide to the eye to highlight the contrast changes of selected molecules by varying the bias voltage (details see text).

To further investigate the charge transfer, we studied the local electronic structure of 1.0 MLE PTCDA on h-BN/Ni(111) by means of STM and STS. Fig. 3(a–c) displays STM images of the same area on the sample but at different bias voltages. At a bias voltage of +1.0 V (tunneling from the tip into unoccupied states of the sample, *i.e.*, investigation of unoccupied molecular states) all molecules show a contrast reminiscent of the lowest unoccupied molecular orbital (LUMO), which consists of two lobes located at the long edges of the perylene core (*cf.*Fig. 1(b)). By decreasing the bias voltage to +0.2 V, we observe that some PTCDA molecules change their contrast to featureless objects (no orbital contrast; see white circle in Fig. 3(b)) while the majority of the PTCDA molecules maintain the LUMO-like contrast. Even more interesting is the fact that also at low negative bias voltages of −0.1 V (tunneling from occupied states of the sample into the tip, *i.e.*, investigation of occupied molecular states) some molecules still show the LUMO-like contrast (see yellow circle in Fig. 3(a–c)). The observation of a LUMO-like contrast at negative bias voltages is a clear indication for a LUMO filling and therefore for a charge transfer.^9^

Furthermore, we recorded STS spectra on different PTCDA molecules (marked with colored crosses in Fig. 3) to investigate the local electronic properties in more detail. For the molecule marked with the blue cross, we observe a feature slightly below and another feature located above the Fermi level (corresponds to a bias voltage of 0 V; see blue curves in Fig. 3(d)), both responsible for a LUMO-like contrast in STM images. This is a clear indication for a splitting of the former LUMO into a singly occupied and a singly unoccupied molecular orbital (SOMO and SUMO) and therefore integer charge transfer. The two slightly different STS spectra are recorded on the perylene core (see dark blue curve in Fig. 3(d)) as well as on the lobe associated with the LUMO (see light blue curve in Fig. 3(d)). In contrast, the molecule marked with the green cross only shows a feature above +0.6 V (see Fig. 3(d)). Since this molecule only exhibits the LUMO-like STM contrast at a bias voltage of +1.0 V, we identify the feature in the STS spectrum with the LUMO of neutral PTCDA. The red STS spectrum was recorded in a hole of the PTCDA layer, where we expect to measure the electronic properties of the h-BN interlayer. Having attributed our STS reference data to charged and neutral PTCDA molecules, respectively, we can identify the charge state of the highly ordered structure visible in the STM image of Fig. 1(b). This image recorded at a bias voltage of +0.1 V shows a LUMO-like contrast which corresponds to charged PTCDA molecules. Therefore, we conclude that the PTCDA anions arrange in an ordered structure. Furthermore, we compare the local electronic properties of the PTCDA molecules in the mono- and bilayer. Fig. S3(a)† displays an STM image with a small ordered bilayer domain. The STS spectrum of a molecule in the second layer (see Fig. S3(b)†) looks quite similar to the STS spectrum of the neutral molecules in the monolayer (see green curve in Fig. 3(d)). We also observe very small features around the Fermi level which resemble the STS spectrum of the anions (see blue curves in Fig. 3(d)). These features may be explained by electrons tunneling into/from unoccupied/occupied states of molecules in the underlying first monolayer.

**Fig. 4:**
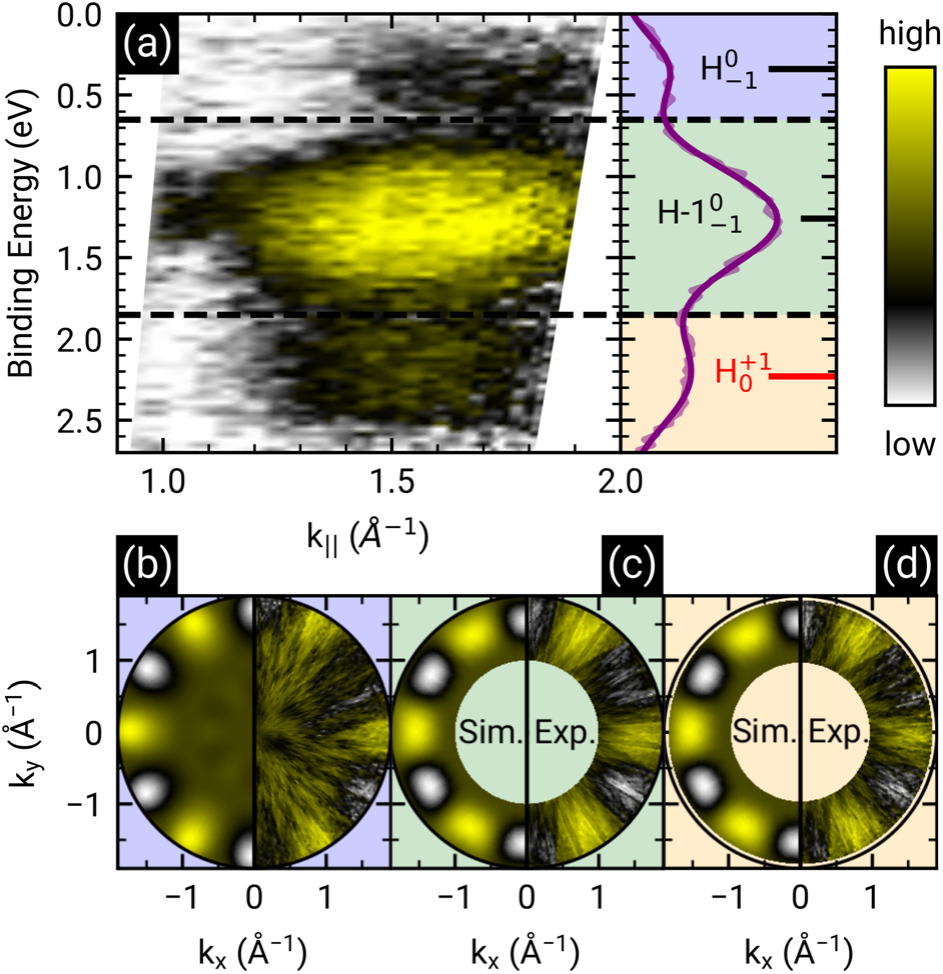
(a) ARUPS measurements of 1 MLE PTCDA on h-BN/Ni(111) along the *Γ*–*K* direction and corresponding energy distribution curve (EDC, purple line). The modified *χ*-contrast is applied to enhance the contrast of the molecular features. The raw data is displayed in Fig. S4 in the ESI.† Three features are visible which are labeled by the corresponding molecular orbital as well as the initial and final states of the probing process as subscript and superscript, respectively, as suggested by Kirchhuebel *et al.*^48^ The positions of the energy levels were determined by fitting three Gaussian functions to the EDC. (b–d) Measured and simulated contrast-enhanced PMMs of the three features visible (marked by different colors). For the simulation of the PMMs, the LUMO (b) and HOMO (c and d) of a single PTCDA molecule in the gas phase is used.

Furthermore, we measured photoemission orbital tomography (POT) on this sample to understand the charge transfer not only locally but also with an area-averaging method. Unfortunately, an identification of the highest occupied molecular orbitals is challenging because of their superposition with the Ni 4d bands which are located directly at the Fermi edge, resulting in a broad background within the POT maps (see Fig. S4 in the ESI†). To overcome this problem, we use the modified *χ*-contrast, which is similar to the normalization procedure used in the analysis of X-ray photoelectron diffraction (XPD) patterns.^49,50^ We define the *χ*-value in the following way:
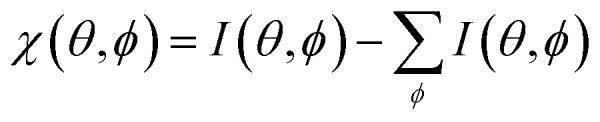


Here, the angles *θ* and *ϕ* correspond to the polar and azimuth angle, respectively. By applying this contrast enhancement we identify three features in the ARUPS measurements (see Fig. 4(a)) along the *Γ*–*K* high symmetry direction of h-BN/Ni(111) at a binding energy of 0.34(2) eV, 1.26(1) eV, and 2.23(2) eV which are marked in blue, green, and orange in the energy distribution curve (EDC). To investigate the origin of these features in more detail we recorded photoelectron momentum maps (PMMs) at the corresponding binding energies. The aforementioned contrast enhancement enables us to compare the measured PMMs with simulations. To this end, we use our structural model proposed earlier consisting of a herringbone arrangement of the PTCDA molecules with two molecules per unit cell (one aligned almost parallel with respect to the lattice vector of the substrate and one rotated by 77° counterclockwise). Additionally, mirror and rotational domains were considered because of the substrate symmetry while neglecting any molecule–molecule interactions or influences of the substrate on the simulated intensity distributions. The comparison of the measured and simulated PMMs shows that the feature at 0.34(2) eV resembles the simulated PMM of the LUMO of PTCDA (see Fig. 4(b)) while the other two PMMs are in good agreement with the simulated PMM of the HOMO (see Fig. 4(c) and (d)). Therefore, we can identify the feature at 0.34(2) eV as former LUMO which is singly occupied (SOMO) due to the integer charge transfer and is now the HOMO of the PTCDA anions. Furthermore, we suggest that the next feature at 1.26(1) eV corresponds to the HOMO-1 of the anions (former HOMO) which is in good agreement with our DFT calculations (see next section). Accordingly, the feature at 2.23(2) eV is assigned to the HOMO of the neutral molecules. In the following we label the molecular features in Fig. 4(a) with the associated molecular orbital as well as the initial state as subscript and the final state as superscript.LEVEL^final state^_initial state_

This notation was introduced by Kirchhuebel *et al.*^29,48^ Since the photoionization of PTCDA in the neutral ground state (initial state = 0) results in singly positively charged molecules (final state = +1), the label of the molecular feature associated with the HOMO of neutral PTCDA is H^+1^_0_. In the same way we can label the molecular feature of the singly negatively charged molecules (initial state = −1) by taking into account that the photoionization causes a depletion of the additional electron and therefore the final state is a neutral PTCDA molecule (final state = 0). Consequently, the label is H^0^_−1_. Using the area of the peaks assigned to molecular features in the EDC (see Fig. 4(a)) corresponding to (H−1)^0^_−1_ (HOMO−1 of the PTCDA anions, former HOMO of the neutral PTCDA) and H^+1^_0_ (HOMO of the neutral PTCDA molecules), we determine the fraction of charged molecules in the monolayer to be approx. 70%.

The similarity between the measured PMMs with a simulation assuming a highly ordered herringbone PTCDA structure seems to be surprising regarding the poorly ordered fashion of the molecular layer in the STM images in Fig. 3. This apparent contradiction can be resolved by a closer inspection of the molecular orientation. Most molecules are aligned nearly perpendicular (*i.e.*, somewhat less than 90°) to their nearest neighbors, while lacking long-range order. Consequently, the PMMs still yield valuable results as this method is highly sensitive to the molecular orientation with respect to the substrate. In particular, the identification of the peak near the Fermi energy as originating from a former LUMO of PTCDA supports our interpretation of an integer charge transfer to a significant part of the molecules. Still, there is the open question about the origin of the charge transfer.

### Density functional theory calculations – elucidating the charge transfer

**Fig. 5:**
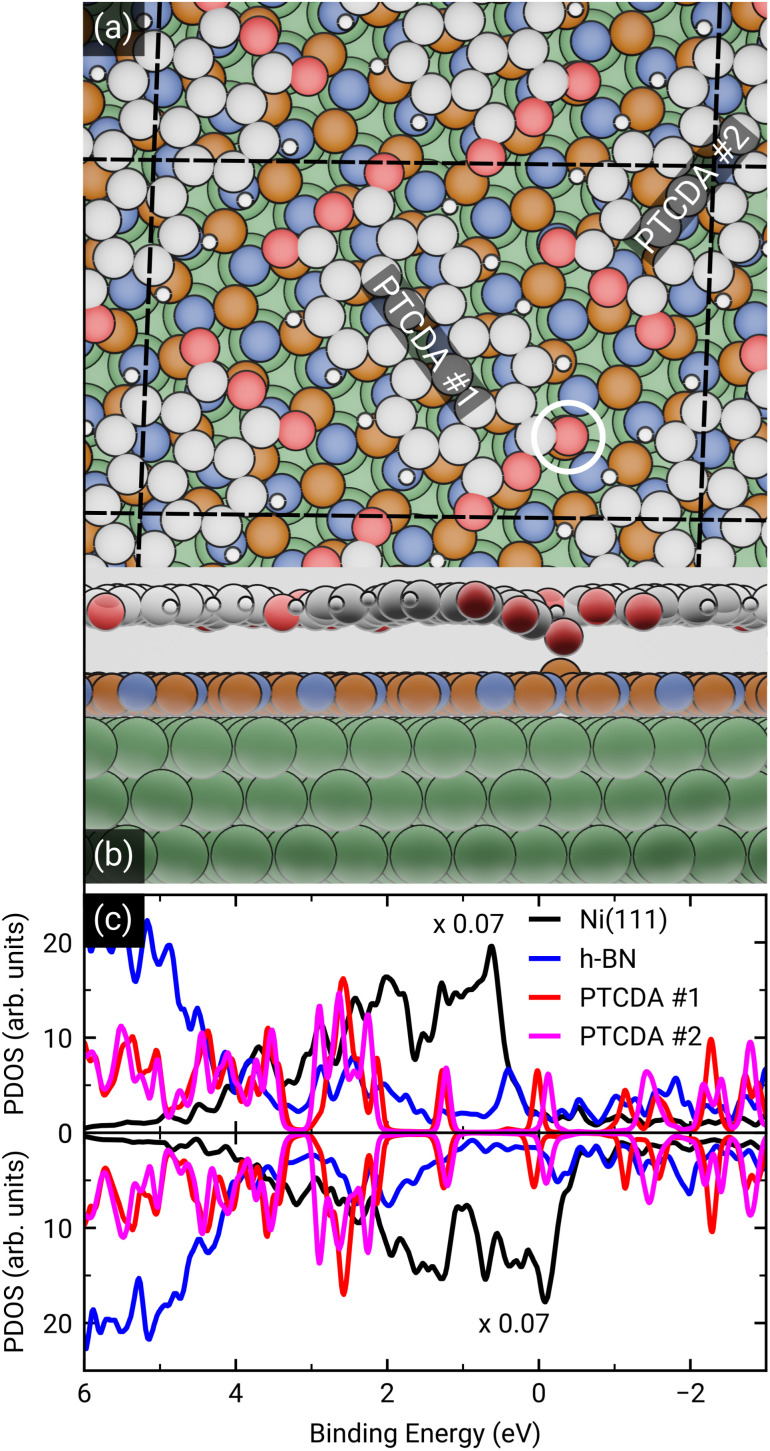
(a) Top and (b) side view of the structural model of PTCDA on h-BN/Ni(111) after geometry optimization. Ni, B, N, C, H and O are represented by green, orange, blue, grey, white and red spheres, respectively. The unit cell consists of two inequivalent molecules which are labeled as PTCDA #1 and PTCDA #2. The side view shows the bond formation of one carboxylic O atom and a B atom of the underlying h-BN. (c) Spin-resolved density of states (DOS) of PTCDA on h-BN/Ni(111) calculated by DFT, with spin-minority DOS being upside down. The LUMO of the two PTCDA molecules is located in close proximity of the Fermi level and seen to be split into a more strongly bound and filled LUMO of PTCDA #1 and less strongly bound LUMO of PTCDA #2.

To dive deeper into the understanding of the charge transfer we performed DFT calculations with periodic boundary conditions to support our experimental results. The starting point for these calculations was the already discussed structural model of PTCDA on h-BN/Ni(111) (see section ‘Thin film growth and structural analysis’). Since the PTCDA molecules adopt a non-commensurate registry with respect to the substrate (h-BN on Ni(111)), we matched substrate and adsorbate lattices by scaling the substrate lattice parameters while keeping the adsorbate lattice fixed to the experimentally obtained values, as detailed in the section ‘Experimental methods and calculation details’. The resulting unit cell is displayed in Fig. 5(a and b). The optimized average adsorption heights of the two inequivalent PTCDA molecules in the unit cell are 3.15 Å (PTCDA #1) and 3.18 Å (PTCDA #2), respectively. These values are in reasonable agreement with the adsorption height of PTCDA on Au(111) (*d*_H_ = 3.27 Å)^51^ and PTCDA on h-BN/Cu(111) (*d*_H_ = 3.37 Å),^52^ which were determined by X-ray standing wave (XSW) measurements. For the latter two cases, a physisorptive behavior was reported.^51,52^ By looking at the side view of the optimized unit cell (see Fig. 5(b)) it can be seen that one carboxylic O atom of PTCDA #1 (see white circle in Fig. 5(a)) bends toward the h-BN layer while the underlying boron atom stands a bit out of the h-BN layer. We determined the energy gain of this attractive interaction by comparing the adsorption energy of each PTCDA in the unit cell to
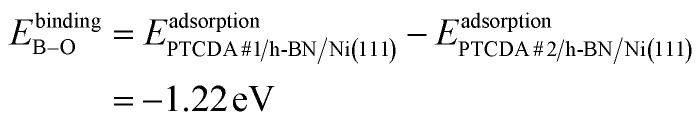


Interestingly, we also obtained experimental evidence for this attractive interaction by analyzing the B 1s core level of 1.5 MLE PTCDA on h-BN/Ni(111) by means of XPS. In Fig. S5 in the ESI† a new boron component is observed on the high binding energy side of the B–N peak of the h-BN layer, which is typical for a boron–oxygen-interaction.^53^ The bending of functional groups of organic singly charged molecules towards 2D materials was also observed for 2,3,5,6-tetrafluoro-7,7,8,8-tetracyanoquinodimethane (F_4_TCNQ, C_12_F_4_N_4_, CAS No.: 29261-33-4) on graphene/Ir(111).^54^

After the geometry optimization the spin-resolved density of states was computed, which shows the position of the lowest unoccupied molecular orbital (LUMO) of the two PTCDA molecules in the unit cell directly located at the Fermi energy (see Fig. 5(c)), resulting in a charge transfer from the substrate to the adsorbate. Furthermore, we obtain a deeper insight into the charge transfer by computing the rearrangement in the charge density (−*e* times the electron density) Δ*ρ*_bond_.^55^Δ*ρ*_bond_ = *ρ*_PTCDA/h-BN/Ni(111)_ − *ρ*_PTCDA_ − *ρ*_h-BN/Ni(111)_.

**Fig. 6:**
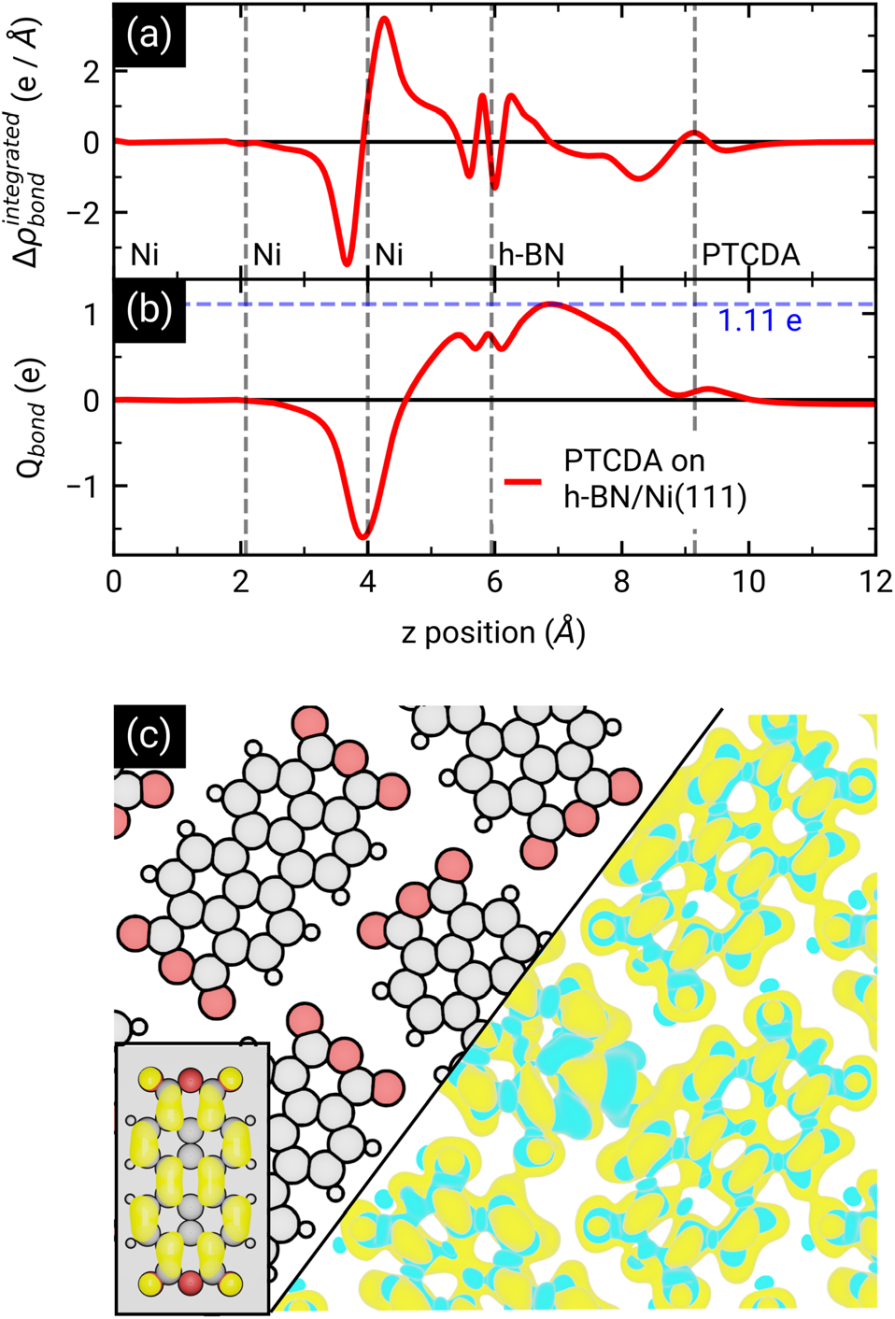
(a) *x*–*y*-integrated adsorption induced charge density rearrangement Δ*ρ*^integrated^_bond_ and (b) total charge transfer *Q*_bond_. Negative (positive) values of Δ*ρ*^integrated^_bond_ correspond to electron accumulation (depletion), while positive (negative) values of *Q*_bond_ implies an electron transfer from left-to-right (right-to-left). Furthermore, we marked the maximum of *Q*_bond_ which corresponds to the amount of transferred charge from h-BN/Ni(111) to the PTCDA layer. In (c) the electron accumulation (yellow, isovalue = −3 × 10^−4^ e Å^−3^) and depletion (cyan, isovalue = 3 × 10^−4^ e Å^−3^) within the PTCDA layer is visualized. The electron accumulation matches very well with the absolute square of the LUMO wavefunction of a single PTCDA molecule in vacuum (see inset).

From this quantity we calculated the total charge transferred up to a certain surface distance *z*, *Q*_bond_(*z*), by integrating Δ*ρ*_bond_ over the *x*–*y*-plane followed by an integration from the bottom of the slab to a certain *z* value:
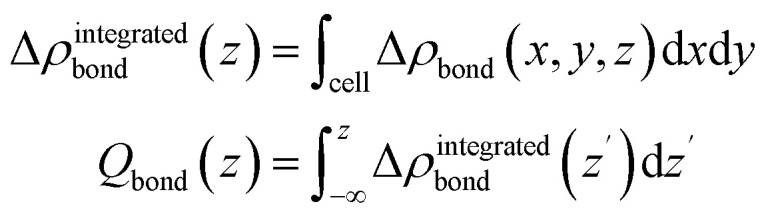


The maximum of *Q*_bond_ corresponds to the amount of transferred charge from the h-BN/Ni(111) interface to the PTCDA layer.^55^ Therefore, we find that the PTCDA layer gains 1.11 electrons per PTCDA unit cell from h-BN/Ni(111). Additionally, the 3D representation of Δ*ρ*_bond_, depicted in Fig. 6(c), shows electron accumulation onto the PTCDA molecules which clearly resembles the LUMO of a single PTCDA molecule in the gas phase (depicted in the inset).

Since both molecules in the unit cell show this LUMO contrast, each molecule gains approx. 0.5 electrons which is rather an indication of fractional than integer charge transfer. Unfortunately, this apparent contradiction with the experimental observation of an ICT cannot be easily resolved due to the constraints imposed by the number of atoms used for the calculations. In our case, even the primitive unit cell is already quite large, which implies substantial computational costs. If one aims at a prediction of integer charge transfer by means of DFT, a significantly larger supercell (containing several primitive adsorbate unit cells and an according number of substrate atoms) would be required, which exceeds the affordable computational costs by far. Moreover, the influence of the exchange–correlation function is another challenge to this endeavour.^7^ Nevertheless, while not able to distribute transferred charge unevenly, our DFT results support the experimental observation of an electron transfer from h-BN/Ni(111) to the PTCDA monolayer and therefore a negative charging of the molecules, as well as the occurrence of B–O bonds.

### Energy-level alignment – reason for the charge transfer

**Fig. 7:**
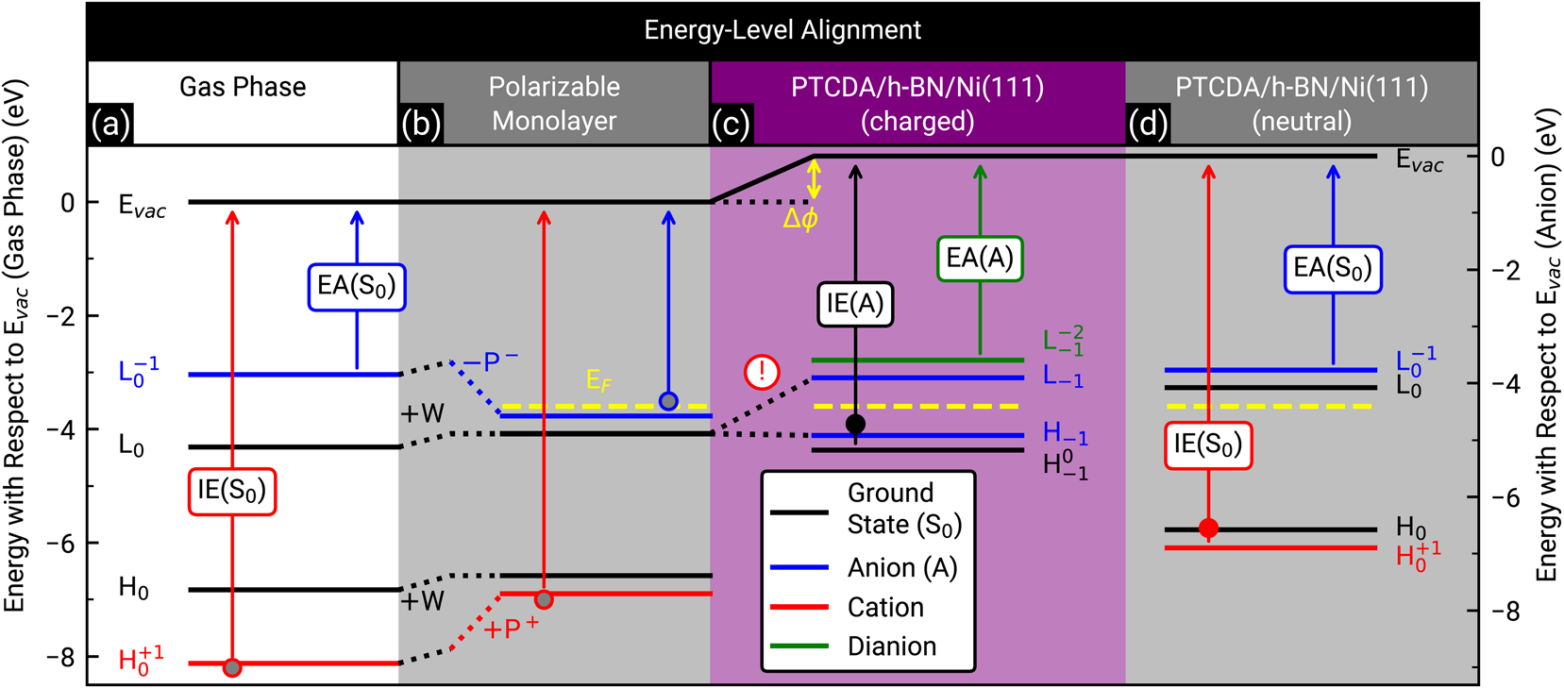
Energy-level diagram of neutral PTCDA molecules in the gas phase (a) and in the monolayer (b) as well as singly negatively charged (anions) (c) and neutral molecules in the monolayer (d). The calculated energy levels are labeled by the corresponding molecular orbital as well as the initial and final states of the probing process as subscript and superscript, respectively, as suggested by Kirchhuebel *et al.*^48^ The energy levels were labeled with H and L for HOMO and LUMO, respectively. The calculated ionization energies (IE(S_0_), IE(A)) and electron affinities (EA(S_0_), EA(A)) are marked by arrows. The electrostatic and induced interactions (W, P^+^ and P^−^) as well as the change of the surface dipole (Δ*ϕ*) are shown as dotted lines and yellow arrow, respectively. The experimentally determined Fermi levels (*E*_F_) of bare h-BN/Ni(111) (b) and PTCDA on h-BN/Ni(111) ((c) and (d)) are displayed as yellow dashed lines. (d) Same as (b), but all energy levels (solid lines) are shifted by Δ*ϕ*. Please notice that in (c) the energy level of the LUMO (L_0_) splits into the energy level of the SOMO (H_−1_) and SUMO (L_−1_) due to the integer charge transfer (see exclamation mark). The ionization energy of PTCDA molecules in the gas phase and in the thin film^56^ as well as the electron affinity in the thin film^57^ are marked by grey, filled red and blue circles, respectively. For comparison, the independently determined ionization energy of PTCDA anions and neutral molecules, which were determined from the ARUPS measurements depicted in Fig. 4(a), are marked as filled black and red circles, respectively. The positions of all simulated and measured energy levels are summarized in Table S1 in the ESI.†

Finally, we discuss the origin of the integer charge transfer by looking at the energy-level alignment. In a previous publication we already showed that spectroscopic features of the molecule tetraphenyldibenzoperiflanthene (DBP, C_64_H_36_, CAS No.: 175606-05-0) in mono- and bilayers on a weakly interacting substrate can be well described by delta self-consistent-field (ΔSCF) calculations of single molecules with a polarizable continuum model (PCM) by considering the initial and final state of the probing process.^29,48^ Here, the energy levels are labeled with the corresponding molecular orbital and the initial as well as final state as subscript and superscript, respectively. In contrast, the energy levels of the unperturbed system only have the initial state as subscript. The latter are represented by the Kohn–Sham eigenvalues of the HOMO and LUMO (H_0_ and L_0_), respectively. Since h-BN is in principle a weakly-interacting substrate similar to graphene/graphite, we adopted the same approach as in ref. 29 for PTCDA on h-BN/Ni(111). We calculated the ionization energy, electron affinity, and ground state energy levels of neutral PTCDA molecules in the gas phase as well as in a polarizable medium with a dielectric constant of 3.05. The results are displayed in Fig. 7(a) and (b). The reduction of the transport gap in the wetting layer in comparison to the gas phase is caused by the interaction of the molecules with the polarizable medium, which can be separated in electrostatic interactions W and induced interactions P. A detailed explanation of the visible energy levels and energy shifts is given in ref. 29. Furthermore, we added the Fermi level in the energy diagram of the wetting layer by using the experimentally determined work function of h-BN/Ni(111) (*E*_F_ = E_Vac_ – *ϕ* = −3.60(2) eV, determined by the secondary electron cut-off (SECO) in UPS). The position of the LUMO of the monolayer (with the polarizable medium, Fig. 7(b)) with respect to the work function of bare h-BN/Ni(111) clearly shows that this level is occupied. Therefore, a charge transfer is expected. This charge transfer leads to Fermi level pinning and a change of the surface dipole Δ*ϕ* = 0.81(4) eV (see Fig. 7(c)), which increases the work function (see Fig. S6 in the ESI†) and therefore shifts the Fermi level to −4.41(2) eV. The energy level of the former LUMO (L_0_) splits into H_−1_ (SOMO) and L_−1_ (SUMO), which are located symmetrically around the Fermi level (see Fig. 7(c)).^10^ Interestingly, the energy level associated with the LUMO of neutral PTCDA molecules matches quite well with the energy level of the HOMO of PTCDA anions which is in line with the expected Fermi level pinning. The energy of the H^0^_−1_ level is determined from our ARUPS measurement in Fig. 4 to be −4.75(3) eV which is even lower than the calculated value of H_−1_.

The investigation of integer charge transfer processes on MgO thin films on Ag(100) shows that a critical work function of the substrate exists under which the integer charge transfer happens, which is also called pinning work function *ϕ*_pinning_.^9–11^ The number of charged molecules scales with the energy needed to raise *ϕ* to *ϕ*_pinning_. In the case of PTCDA on h-BN/Ni(111), the pinning work function is equal to 4.41(2) eV which is in good agreement with the pinning work function observed for 1 MLE PTCDA on ZnO.^8^ Since we observe charged and neutral PTCDA molecules on h-BN/Ni(111) we conclude that Δ*ϕ* is not large enough to charge all molecules. We will explain the energy-level alignment of neutral molecules in the following. The charge transfer shifts the vacuum level to higher energies, and therefore also the energy levels of the neutral molecules are shifted in the same way (vacuum level alignment) while the position of the Fermi level stays the same (see Fig. 7(d)). Consequently, L_0_ has now an energy which is above the Fermi level and therefore no charge transfer occurs. Furthermore, we can determine the energy H^+1^_0_ from our ARUPS measurement (see Fig. 4(a)) which is in reasonable agreement with our DFT calculations (see Fig. 7(d)). The positions of all calculated and measured energy levels are summarized in Table S1 in the ESI.† Furthermore, we added the experimentally determined ionization energies of PTCDA in gas phase and thin films^56^ as well as the vertical electron affinity of PTCDA thin films, which we calculated from the low energy inverse photoelectron spectrum in ref. 57 by fitting a Gaussian function with a linear background and using the corresponding center energy of the peak. Small deviations of the thin film values are expected because the dielectric constant could be different for the monolayer and larger film thicknesses.

In summary, the low work function of h-BN/Ni(111) triggers the integer charge transfer, and the former LUMO splits into a SOMO and SUMO. However, the work function is not low enough to charge all PTCDA molecules, therefore a superposition of singly negatively charged and neutral molecules is observed. The work function is also the reason why integer charge transfer is not observed for PTCDA on h-BN/Pt(111) and on BlueP/Au(111), since it is higher (*ϕ*_h-BN/Pt(111)_ = 4.9 eV (ref. 58) and *ϕ*_BlueP/Au(111)_ = 5.20(2) eV (ref. 59)) than the pinning work function of PTCDA (*ϕ*_pinning_ = 4.41(2) eV (this work)).

In the end, we discuss how we interpret the simultaneous growth of anionic and neutral PTCDA molecules on h-BN/Ni(111) (compare Fig. S1 in the ESI†). This scenario has already been reported by Hofmann *et al.* for TCNE on NaCl/Cu(100) by using DFT calculations.^7^ They found that the first TCNE molecule adsorbed on the substrate gets negatively charged and becomes an anion. This creates a charge exclusion region around the molecular anion. Molecules adsorbed in this region remain neutral because the interface dipole is not large enough to charge all molecules in the monolayer. Hofmann *et al.*^7^ also observed a linear increase of the interface dipole for 2D cluster growth (starting from different crystallization seeds) in the submonolayer regime until the monolayer is complete. This implies that anions and neutral molecules adsorb simultaneously on the surface. 2D cluster growth is reasonable for PTCDA due to the strong intermolecular interaction, and has been reported for submonolayer coverage on Au(111).^60^

### Conclusion

In summary, we investigated the integer charge transfer of a highly ordered monolayer of PTCDA on h-BN/Ni(111). We observe single integer charge transfer of organic molecules by measuring the optical response of PTCDA anions using DRS during the growth process. Furthermore, the PTCDA monolayer on h-BN/Ni(111) consists of both, charged and neutral molecules. The integer charge transfer was further rationalized by STS and POT measurements. STS reveals a density of states around the Fermi level and even a LUMO-like contrast in the occupied states for some PTCDA molecules. It was also possible to distinguish charged and neutral molecules due to the different STM contrasts and fingerprints in the STS spectra. By comparing the recorded contrast-enhanced PMMs with the simulation using the LUMO and HOMO of PTCDA, we could identify the SOMO of the PTCDA anions as well as the HOMO of the neutral molecules. In addition, based on the angle-resolved UPS measurements of a PTCDA monolayer on h-BN/Ni(111), the fraction of the anions was determined to be approx. 70%. The charge transfer is also supported by our DFT calculations. The simulations show the position of the LUMO located at the Fermi level. From the adsorption-induced charge density rearrangement we calculated the amount of transferred electrons from the h-BN/Ni(111) interface to the PTCDA layer to be approx. one electron per unit cell which consists of two PTCDA molecules. Lastly, we discussed the energy-level alignment in terms of the model introduced by Kirchhuebel *et al.*^29,48^ The integer charge transfer is only possible due to the weak molecule–substrate interaction of PTCDA on h-BN/Ni(111) where essentially no overlap between molecular orbitals and metal states exists (otherwise, fractional charge transfer would be observed) and driven by the low work function of h-BN/Ni(111). This leads to a modification of the optical and electronic properties of the formerly neutral PTCDA.

Other 2D materials such as MoS_2_ can also be used to decouple organic molecules from the metal surface,^61^ but the work function of such layers is usually too large^62^ (ranging from approx. 4.4 eV to approx. 5.4 eV), and the choice of molecules which exhibit an integer charge transfer is limited due to the requirement of a very high electron affinity (high pinning work function) of the adsorbed molecules^63^ (even higher than the electron affinity of PTCDA). In contrast to these 2D materials, h-BN on Ni(111) exhibits a rather low work function of 3.60(2) eV which increases the variety of molecules for which integer charge transfer is expected. In addition, this h-BN layer is atomically flat and therefore supports the growth of highly ordered 2D molecular layers.

## Author contributions

M. S. and M. G. performed the DRS and STM measurements. M. S. and F. O. measured the LEED images. M. S. conducted the PES and STS measurements. A. B. and G. F. performed the DFT calculations of the periodic surface slab. M. S. performed the DFT calculations of single molecules. M. S. conducted the data analysis, literature research as well as the manuscript writing with input from all co-authors. All authors reviewed the final manuscript.

## Conflicts of interest

The authors declare no competing financial interest.

## Supplementary Material

NA-006-D4NA00462K-s001

## Data Availability

The data shown in the figures of the article and the corresponding ESI file† are available on request from the corresponding author.
