# Supplementary material for: Triggered integer charge transfer: energy-level alignment at an organic-2D material interface
Source: Nanoscale Adv. 2024 Jul 30;6(19):4932–43. doi: 10.1039/d4na00462k (PMC11320374; doi:10.1039/d4na00462k)
Supplement: NA-006-D4NA00462K-s001 [file NA-006-D4NA00462K-s001.pdf]

Supplementary Information (SI) for Nanoscale Advances.
This journal is © The Royal Society of Chemistry 2024

Supplementary Information:
Triggered integer charge transfer: energy-level alignment at an organic-2D material interface

Maximilian Schaal[1], Anu Baby[2,3], Marco Gruenewald[1], Felix Otto[1], Roman Forker[1], Guido Fratesi[4], and Torsten Fritz[1]

[1]*Institute of Solid State Physics, Friedrich Schiller University Jena, Helmholtzweg 5, 07743 Jena, Germany*

[2]*Department of Materials Science, University of Milano-Bicocca, Via R. Cozzi 55, 20125 Milano, Italy*

[3]*STMicroelectronics, Via Tolomeo 1, 20010 Cornaredo, Italy*

[4]*ETSF and Dipartimento di Fisica "Aldo Pontremoli", Università degli Studi di Milano, Via Celoria, 16, 20133 Milano, Italy*

Layer thickness determination by DRS measurements

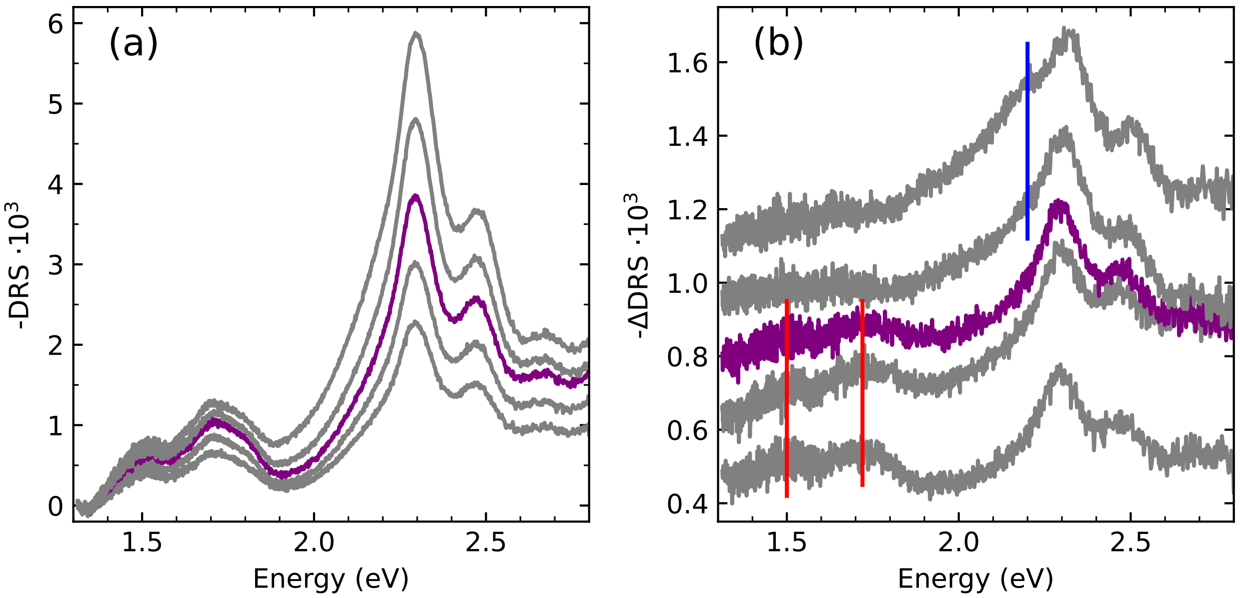

Figure S1 (a) DR and (b) ΔDR spectra of PTCDA on h-BN/Ni(111). The spectrum of the first monolayer equivalent is marked in purple. We added vertical lines as guides to the eye to illustrate the change of the optical properties of the organic molecules.

LEED Measurement of 1.0 MLE PTCDA on h-BN/Ni(111)

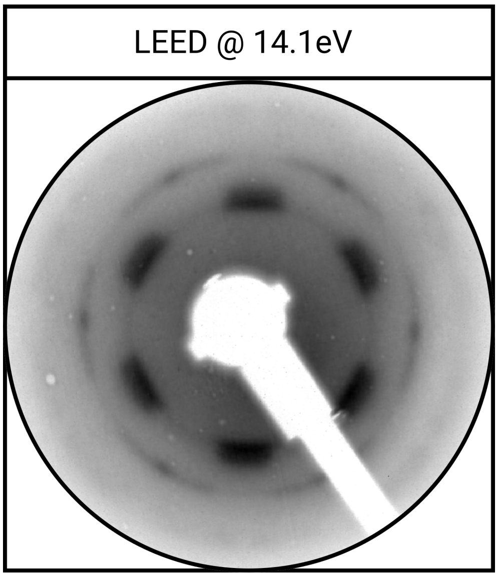

Figure S2 LEED image (logarithmic intensity scale, contrast inverted) of 1.0 MLE PTCDA on h-BN/Ni(111).

STM and STS of PTCDA Molecules in the Second Layer

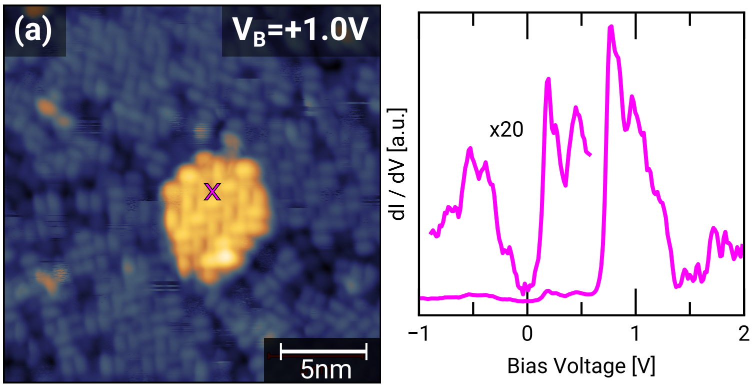

Figure S3 (a) 20 nm × 20 nm STM image of a small bilayer domain of PTCDA on h-BN/Ni(111) measured with a bias voltage of +1.0 V and a tunneling current of 30 pA. The magenta cross in the STM image visualizes the location of the performed STS measurements which is displayed in (b). A detailed discussion is given in the main text (section 'Electronic Structure - Manifesting the Charge Transfer').

Raw Data of Angular Resolved Ultraviolet Photoelectron Spectroscopy and Photoelectron Orbital Tomography

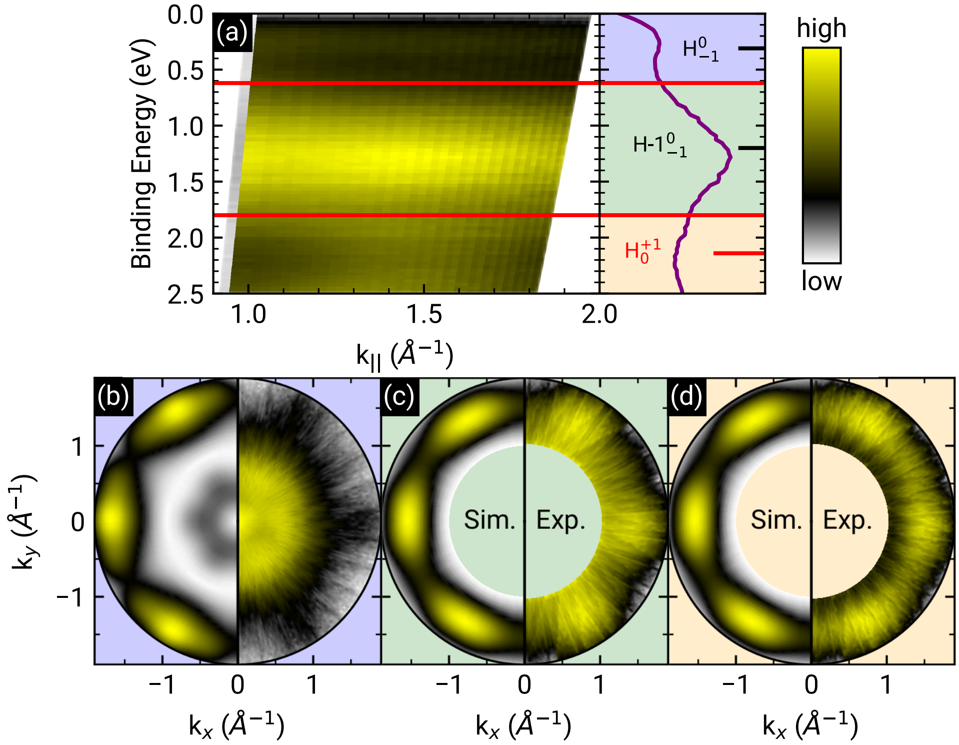

Figure S4 (a) ARUPS measurements along the Γ-K direction and corresponding energy distribution curve (EDC, purple line). The three features also marked in Fig. 4 in the main text are labeled by the corresponding molecular orbital as well as the initial and final states of the probing process as subscript and superscript, respectively, as suggested by Kirchhuebel et al.[1]. (b-d) Measured and simulated PMMs of the three features visible (marked by different colors). For the simulation of the PMMs the LUMO (b) and HOMO (c, d) of a single PTCDA molecule in the gas phase is used.

Experimental Evidence for a B-O Bond

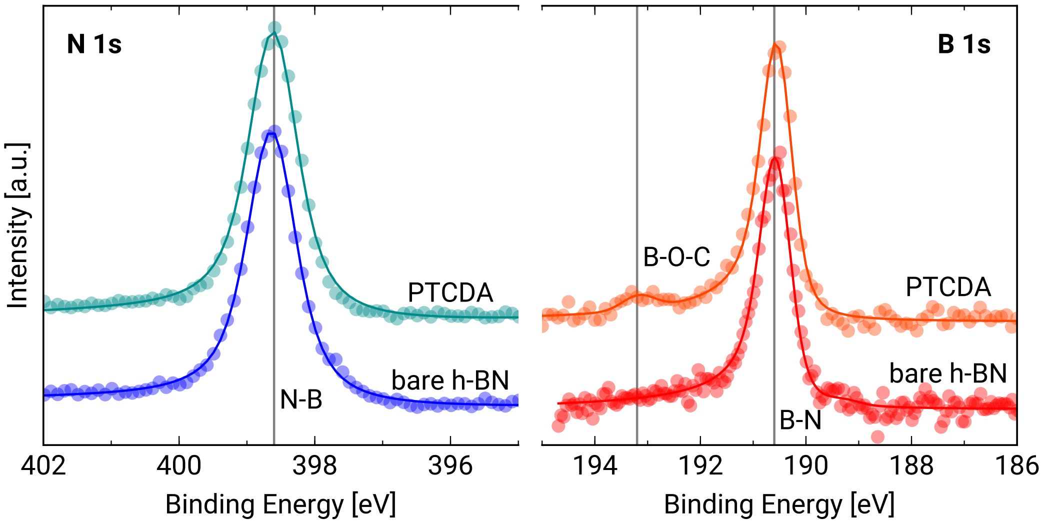

Figure S5 B 1s and N 1s core level spectra of bare h-BN on Ni(111) and 1.5 MLE PTCDA on h-BN/Ni(111). The line shapes are fitted by a Voigt function in combination with an active Shirley background. After the adsorption of PTCDA on h-BN/Ni(111) a new boron component arises at a binding energy of 193.2(1) eV, which is taken into account in the line shape analysis by adding an additional Voigt function.

Shift of the Secondary Electron Cut-Off due to the Charge Transfer

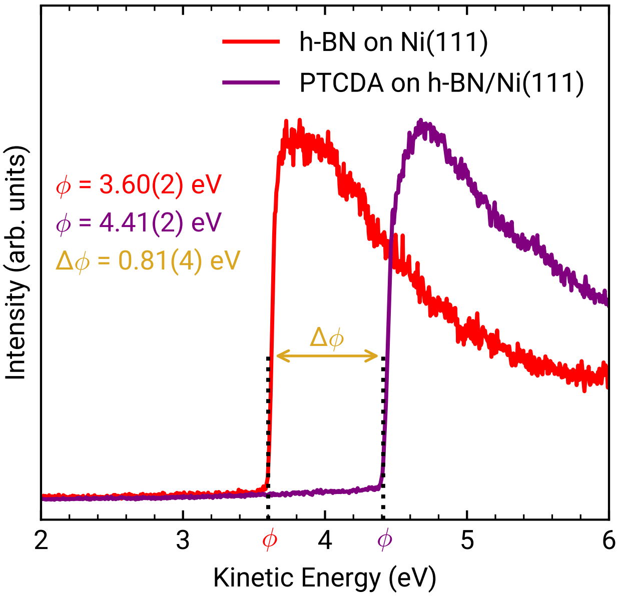

Figure S6 Secondary electron cut-offs (SECOs) of h-BN on Ni(111) and PTCDA on h-BN/Ni(111). The work function ϕ of each sample was determined by the onset of the SECO. The change of the surface dipole $\Delta\phi$, which is caused by the charge transfer, was calculated by the difference of the work functions.

Quantities of the Energy-Level Alignment Diagram

Table S1 Calculated and measured energies of the energy level as depicted in Fig. 7.

	L_0^{-1} (eV)	L_0 (eV)	H_0 (eV)	H_0^{+1} (eV)	E_{trans} (eV)
**Gas Phase (Neutral)**	-3.00	-4.31	-6.83	-8.12<br>-8.20(3)[a]	5.12
**Monolayer (Neutral)**	-3.72<br>-3.51(1)[b]	-4.08	-6.58	-6.90<br>-7.0(2)[a]<br>-6.64(4)[c]	3.18
	**L_{-1}^{-2} (eV)**	**L_{-1} (eV)**	**H_{-1} (eV)**	**H_{-1}^{0} (eV)**	**E_{trans} (eV)**
**Charge Transfer & Fermi Level Pinning**	-3.63	-3.91	-4.93	-5.13<br>-4.75(4)[c]	1.50

[a] Ref. 2

[b] Ref. 3

[c] This work: ARUPS measurement (see Fig. 4(a) in the main text)

References:

[1] T. Kirchhuebel, O. L. A. Monti, T. Munakata, S. Kera, R. Forker and T. Fritz, *Phys. Chem. Chem. Phys.*, 2019, **21**, 12730–12747.

[2] J. Sauther, J. Wüsten, S. Lach and C. Ziegler, *J. Chem. Phys.*, 2009, **131**, 034711.

[3] H. Yoshida, *J. Electron Spectrosc. Relat. Phenom.*, 2015, **204**, 116–124.
